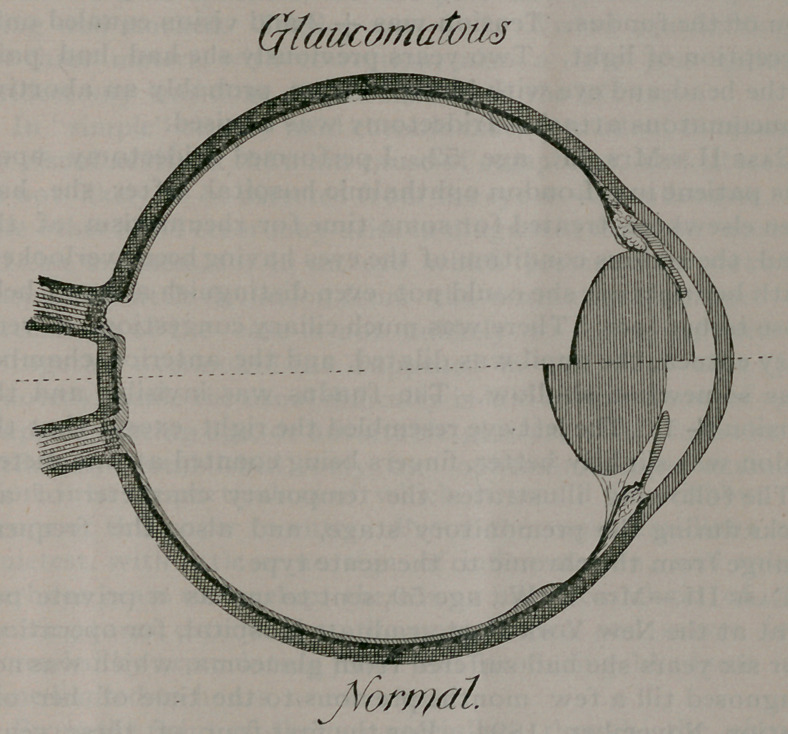# Glaucoma in Relation to General Practice

**Published:** 1896-08

**Authors:** Alex. W. Stirling

**Affiliations:** Atlanta, Ga.


### Southern Medical Record.

A MONTHLY JOURNAL OF MEDICINE AND SURGERY.

VOL. XXVI. ATLANTA, GA., AUGUST, 1896. No. 8.

### Original Articles.

### GLAUCOMA IN RELATION TO GENERAL PRACTICE.*

BY ALEX. W. STIRLING, M. D. (HONORS), M. B. AND C. M. (EDIN.) D. P. H. (LOND.), ATLANTA, GA.

GENTLEMEN: Glaucoma is to the ophthalmic specialist one of the most interesting of diseases. It is interesting to him because, from the remotest times into which the history of medicine can carry us down to the present day, it has been a subject of difficulty and discussion; because there is probably no other ocular disease, a proper understanding of which has been so dependent upon a clear grasp of true physiological processes within the eye, and whose development has followed so closely upon these; because it is a morbid process of such relentless nature that, once established, if uncombated, it seldom or never relinquishes its deadly progress until the eye, and in the great majority of cases, both eyes, are quite disorganized, and, as if not content with their destruction, it frequently continues to torment its victim with such pain that he urgently calls for removal of the globe.

Glaucoma interests the specialist also because in one of its forms it is sometimes very difficult of diagnosis; because frequently so much can be done toward cutting short its course, or even saving the health of the eye; and also because it is one of the commonest of dangerous ocular diseases, compris-

*Read before Medical Association of Georgia, April, 1896.

ing, according to a high authority, as much as one per cent. of all ophthalmic troubles.

But to give the impression that this interest is necessarily confined to specialists, would be to controvert the very object of this paper, which is a humble attempt to increase an interest already felt, or create one if still unfelt, in the minds of such of the members of this society as may not have given any important degree of their attention to the subject of ophthalmic pathology. While I believe that every case of glaucoma requires the most skilful advice that can be obtained, it is first and foremost a disease which comes within the province of the general practitioner, and with him lies frequently the ultimate safety or destruction of the eye involved. That is undoubtedly the case, because, as in all diseases whose progress is attended by unalterable organic change, the early period, that in which the general practitioner is usually consulted, is the hopeful one for treatment. And that this subject is worthy of being brought up in such general meetings as the present, I am sure most ophthalmologists will agree, for I know that it has been the experience of many others, as it has been mine, to meet with no inconsiderable number of cases of glaucoma in which its peculiar symptoms have led astray both patient and practitioner to such an extent that, in consequence, an eye has been either entirely lost, or, at least, has greatly suffered. And very little need be said in order to show how easy it is to fall into such error, as well as how simple it is to correctly diagnose glaucoma, provided that its possible existence be kept before the mind in the presence of certain symptoms which tend rather to draw the attention from, than to, the eye.

We will leave out of account for the moment the more chronic forms of the disease, in which, for reasons to be mentioned later, the eye is less likely to be overlooked than in the more acute. I would here direct your attention to the sketch, which represents half a glaucomatous placed alongside half a normal eye.

In the eye we have an organ whose sensitive nerve supply constitutes a portion of a nervous trunk which sends branches to a great part of the same side of the head and face, and has close central relationship with other nerves proceeding to different parts of the body. Given an irritation of sufficient intensity of the ocular fibres of the trigeminus, and we are very likely to find its action transferred to some or all of the remaining sensory fibres of the nerve, with the result that the pain, originating in reality in an unusual situation, the interior of the eye, but felt with equal severity in situations much more commonly painful, teeth, forehead and ear, is referred in all probability by the patient to an attack of toothache, rheumatism, neuralgia, even to erysipelas, or when associated, as not unfrequently happens, with vomiting, to migraine or sick headache. Such illusions are still more probable, and the physician himself less likely to correct the patient, when the pain extends still farther and is felt in the region of the shoulder. It would be wearisome to multiply here examples of those unfortunate cases, but one or two from among those which I have personally encountered will sufficiently serve the purpose.

CASE I.—Mrs. B. came to my clinic at the Post-graduate Hospital, New York, in October, 1894, with the complaint that she had for the previous six weeks suffered intensely from what she had been given to understand was neuralgia of the right side of the face and head, and also that the right eye had lost its vision. The eye, on examination, showed ciliary injection, haziness of the cornea, shallowed anterior chamber, dilatation and irregularity of the pupil. The opacity of the media interfered with a satisfactory examination of the fundus. Tension was + 2 and vision equaled only perception of light. Two years previously she had had pain in the head and eye with blurred vision, probably an abortive glaucomatous attack. Iridectomy was advised.

CASE II.—Mrs. M., age 52. I performed iridectomy upon this patient in a London ophthalmic hospital, after she had been elsewhere treated for some time for rheumatism of the head, the serious condition of the eyes having been overlooked. With her *right eye* she could not even distinguish a hand held close to her face. There was much ciliary congestion, a very hazy cornea, the pupil was dilated, and the anterior chamber was somewhat shallow. The fundus was invisible and the tension + 1. The left eye resembled the right, except that the vision was slightly better, fingers being counted at one meter.

The following illustrates the temporary character of attacks during the premonitory stage, and also the frequent change from the chronic to the acute type:

CASE III.—Mrs. D. W., age 50, sent to me as a private patient at the New York Post-graduate Hospital, for operation. For six years she had suffered from glaucoma, which was not diagnosed till a few months previous to the time of her operation, November, 1894. For the first four of these years she suffered at intervals, when tired, from pain over both brows and in the eyes, with halo around the lamp in the evening, which symptoms were cut short by food or rest. At the end of the fourth year these symptoms increased in severity, and twelve months later an oculist prescribed glasses for her. During the last few months she had been using eserine, prescribed by a surgeon who had diagnosed glaucoma, but a week before her arrival at the hospital she had experienced an acute attack in both eyes. My notes at the time of entrance state that in the right eye her vision, with correction, a low *plus* cylinder, equaled half the normal, and the field was small. Recently the tension had come down to normal and the anterior chamber was shallow, the pupil was contracted by eserine and the media were so obscured that the iris could not be well seen. The left eye was much congested, the cornea partially opaque, the anterior chamber shallow, the pupil dilated in spite of eserine, the lens partially opaque, some vitreous opacities, the disc pale and glaucomatous cupping well marked. Tension was + 1. Vision equaled fingers at three meters, with correction, also a low *plus* cylinder. Iridectomy was done at this time upon the left eye.

In "simple" or very mild chronic cases a gradual diminution in visual acuity is the main cause of complaint, and attention is not likely to be diverted from the eye as in acute cases. On the other hand error may arise in diagnosis, first, on account of this mildness, for in an eye which presents to outward inspection little deviation from the normal, the proper appreciation of the case is not unlikely to be postponed or altogether overlooked (as happened in the third case just quoted), while the usual difficulty is laid at the door of perhaps advancing age, or unsuitable glasses. The truth can be definitely established only by ophthalmoscopic examination, into the particulars of which it would be without the scope of this paper to enter. From the most insidious and quietest, with little or no sign of inflammation, and taking perhaps years to develop, there is an unbroken chain of connecting cases, extending to the most sudden and severe form attended by excruciating pain, and producing blindness in a time limited to hours, or even less.

From the point of the non-specialist, though the chronic form should also be borne in mind, the typical symptoms of glaucoma for which he should be on the outlook are chiefly, besides impaired vision, pain in the eye, head and face; halos or rainbows seen by the patient round artificial lights; a variable amount of congestion of the eye and of haziness of the cornea; a diminution in the distance between the cornea and the iris as compared with the healthy eye; an enlarged pupil comparatively irresponsive to the stimulus of light, and in which may often be observed the greenish coloration, not, however, peculiar to glaucoma, but from which the disease originally took its name; and most important of all, a heightened tension of the globe felt when it is palpated as for an abscess, through the upper lid, between a finger of either hand, the patient looking down, and distinct in direct ratio to the acuity of the attack. A difference in the tension of the eyes of any individual is always pathological. It is measured by Bowman's signs + or — 1, 2 or 3, according to the augmented hardness or softness of the eye. To this last symptom I should like to direct special attention, and urge that every medical man should make himself familiar—a simple matter—with normal, in order to appreciate abnormal, tension.

It would take too long and would be out of place to discuss in this paper with any degree of fullness what has been written in explanation of the relationship between the high tension glaucoma and the other symptoms. Let it suffice to say that it is the belief most generally accepted, and to my mind on the best of grounds, that, even in chronic cases in which it is sometimes hard to distinguish any increase of intraocular pressure, the latter is yet the *fons et origo* of all that makes glaucoma, and in its absence it is highly improbable that the complete picture of glaucoma is ever attained.

I hope I may be excused if I take the liberty of briefly reminding you of the salient features of the ocular lymph circulation, without a knowledge of which it would be hopeless to expect to understand the pathology of this disease, than which there is no other more striking and visible example of physiology gone wrong.

The eye might be said to consist of a principal and three subordinate systems; the first—for which the others exist—the nervous apparatus prepared for the reception of light and the conduction of the resulting impressions to the brain; the second, which can to a considerable extent be dispensed with when artificial aid is substituted—a partially mobile focusing arrangement; the third, to nourish, and the fourth, to protect the whole.

The first is the sensitive tentacle thrust from the anterior surface of the brain, and round which is gradually built up the remainder of the eye, on whose interior this nervous process is spread out fine as the complicated structure of the retina or innermost of the three layers which together compose the outer framework of the globe.

The cornea, or anterior transparent part of the eye, and the lens together make up the second system. The lens lies just behind the iris and a little posterior to the level of the corneo-scleral junction. In early fœtal life it is spherical and fills the whole interior of the globe to whose circumference it is slung by the suspensory ligament. As the walls of the eye expand more rapidly than does the lens, the latter is compressed by the dragging of the ligament until it ultimately assumes its well-known form. But it does this under compulsion, for upon the slightest slackening of the ligament, and just in proportion to that, due to the action of the accommodation or ciliary muscle, it tends to assume its original form and the refraction of the eye becomes *pari passu*, accordingly increased.

Although the iris falls anatomically within the nourishing system, physiologically it has little connection with it, and is useful mainly as a movable curtain to reflexly regulate the supply of light to the retina.

The third, or nourishing system, is the continuation backward of the iris between the retina and the external membrane or sclerotic, in the form of ciliary body for a few millimeters, and then of the choroid. Both of these are, for the most part, composed of blood-vessels, the capillaries lying next the retina.

Protection is afforded to all by the density and toughness of the sclerotic, of which the cornea is a modification, and in this connection also a part, while from within, the proper shape and tension of the globe are maintained by the presence of the aqueous in front of the lens, and the more gelatinous vitreous behind it.

This brings us to the connecting link between physiology and the pathology of glaucoma. It is evident that just a certain quantity of fluid—no more and no less—will suffice to keep the tension normal; too little, and the eye will tend to become soft, wrinkled, and collapsed; too much. and the delicate structure of its interior will be stretched, compressed, and injured.

A few words must be said concerning this occasional variability in tension. It is in the ciliary region that the fluid enters the vitreous, brought there by the blood in the ciliary arteries, which is returned along with that to the iris and choroid almost entirely by veins which converge to some five main channels called vortex veins, which pass obliquely backward through the sclerotic, a little behind the equator. This lymph, circulating in part slowly through the vitreous, passes in the main around the lens, through the meshwork of its suspensory ligament between the lens and iris, into the angle of the anterior chamber, which it leaves by filtration into a canal running throughout the circle of the cornea, and anastomosing with veins by which it is carried directly from the eye near the corneo-scleral junction. A little fluid also leaves in the region of the optic nerve.

In considering the question of intraocular pressure it is at once evident that an excess of fluid may exist within the eye because too much has entered it, because too little has left it, or from a combination of these causes. Mackenzie, of Glasgow, was the first to observe increase of tension, and in 1830, he showed its connection with glaucoma in his famous work on diseases of the eye. He thought the tension was due to a choroiditis from which fluid was exuded into the vitreous; and he is to be excused for his error, because at that time the ophthalmoscope was still unthought of, and because it is one shared in even now in certain quarters. Von Graefe, who did so much for the cure of the disease, but never could tell how his operation acted, held that, in all probability, there existed an exudation resulting from what he called a "serous choroiditis," because he never could discover with the ophthalmoscope sufficient evidence of true ordinary choroiditis or of any other likely cause for the condition. Donders, stimulated thereto by the then novel researches of others in connection with secretion from glands, considered the supposed exudation to be due to a neurosis causing dilatation of choroidal vessels.

All these theories may now be set aside, because we know that an excessive exudation alone can have only temporary effect upon the tension, and for the following reason: If the amount of fluid be reduced below the normal, the resulting loss of pressure on the choroidal vessels encourages their dilatation and an excessive exudation of fluid through their walls, while, for like reason, less fluid leaves the eye, till the normal is again reached.

In the same automatic manner an excess of vitreous fluid compresses the choroidal vessels and causes abnormal rapidity of filtration from the corneo-iritic angle till the advent of the ordinary tension. It is only, therefore, when combined with retention, that excessive secretion can permanently heighten the pressure, while retention alone is sufficient. But simple as it may appear at the first glance, the explanation of the true origin of this retention has been one of the most debated of ophthalmic questions. Theory is easy, but must be reconciled with clinical experience and pathological anatomy. Theoretically, fluid may be retained by abnormality in the region of the optic nerve, of the vortex veins, or of the corneo-iritic angle. In a mere survey like this the optic nerve route may be left out of account, because, however interesting as a possible focus for the glaucomatous process, the arguments advanced in favor of it as the true one sadly lack that basis of observed fact upon which alone one can safely build. The vortex veins present an excellent opportunity for theory, which has not been forgotten. Any interference with their lumen would, of necessity, result in some degree of stasis and swelling in the choroid, with possibilities of such other secondary abnormalities as open up a tempting field for theorizing, of which several excellent ophthalmologists have taken full advantage. Birnbacher and Czermak, for example, have described a few cases in which peri- and endo-phlebitis have been observed in excised glaucomatous eyes. I published,* some years ago, a criticism of these, along with an account of the condition of the vortex veins of twenty eyes excised for primary glaucoma, and I shall go no further into the matter here than to say that in only three of them were the veins abnormal, and in none of these was there any condition which appeared likely to have resulted in glaucoma. Priestley Smith came to a similar conclusion in connection with thirteen eyes which he examined with a like object. One

*"An Inquiry into the conditions of the Vortex Veins in Primary Glaucoma." Royal London (Moorfield's), Ophthalmic Hospital Reports, vol. xiii. part 4, 1893, p. 419.

can therefore conclude with a considerable degree of certainty, that inflammatory changes in the vortex veins, if they have any connection with glaucoma, result from, rather than produce it.

There remains now only the corneo-iritic angle, and here we meet with conditions always present in glaucoma, except in rare cases which are susceptible of explanation. Upon these changes, and chiefly through the labors of Knies, Weber, and Priestley Smith, an excellent theory has been built up capable of explaining the various symptoms of the disease as well as the effects of treatment. The first step towards this solution was the observation that the filtration area of the corneo-iritic angle is generally blocked in more or less of its circumference by peripheral apposition, or adhesion of the iris and cornea, due, according to Knies, to inflammatory exudation in that region, and, according to Weber, to swelling of the ciliary processes, which push the iris up against the cornea. Weber's idea seems to have a better foundation than that of Knies, and almost certainly explains a certain number of cases. Priestley Smith, by an exhaustive series of laborious experiments, greatly modified the whole theory in that he placed the initial obstruction, not at the corneo-iritic angle itself, but in the peri-lenticular space. He proved that the lens, an epiblastic structure, peculiar in being shut off from communication with the outer world, grows by the superposition of layer upon layer from the outside, none of which can be cast off, as happens, for instance, in the skin, the lens, therefore, continuing to increase in size and weight throughout life, so that at sixty-five it is one-third greater in volume and in weight than at twenty-five. He has also shown that glaucoma is more common in small eyes than in large, and that small size of the globe by no means implies a proportionately small size of the lens. Advanced age, then, as we all know, and smallness of the eye are separately predisposing causes of glaucoma, and in the large majority of cases these are found combined. This is explained by the fact that in these cases the normal peri-lenticular space is trespassed upon from the one side by the lens, the result of age, and from the other by the ciliary processes, the result of the meager diameter of the globe. Slight congestion in the ciliary region, such as might result from ill health, tiredness, strain, etc., which, as a matter of fact, so often precede glaucoma and which, in better circumstances, would probably pass away without evil result, might, in such an eye as that described, by approximating lens and ciliary processes, block the peri-lenticular space, and shut up the fluid behind it, where an accumulation will take place producing the visible anterior displacement of lens and iris so nearly universal in glaucoma.

This initial apposition of iris and cornea may set up after a varying time an inflammatory adhesion between them, which interferes with the at one time possible return of the iris to its normal position.

This is a rough outline merely of the more important of numerous theories of glaucoma etiology, sufficient to indicate in what direction one now looks for an explanation of its peculiar symptoms, and I trust not altogether uninteresting even to him whose work does not lie in that direction. But at the same time that the above theory is probably applicable to most cases of the ordinary senile form, it should be carefully borne in mind that glaucomatous destruction will result from increase of tension, however produced, and that there are various other means by which the iritic anglet may be closed, as for example in certain secondary cases, and in that interesting disease, the glaucoma of childhood, which there is not time here to discuss.

It will occupy only a moment to consider how fully an increase of intraocular pressure can account for all the symptoms. Pressure on the sensory nerves of the eye and transference of the sensation to other branches of the same nerve or to other nerves, cause the pain in the eye and elsewhere. Pressure produces the halos or rainbows through œdema of the cornea, the result of interference with circulation, which corneal haze, along with the effects of pressure on the main trunk of the optic nerve, as well as on the nervous and vascular structure of the retina and choroid, accounts for the diminished visual acuity. Acting on the vortex veins in their oblique passage through the ocular walls the pressure produces the pink zone of enlarged, vicariously employed vessels, seen around the cornea. We have noticed how the anterior chamber is shallowed, an explanation which holds good also for the large and more or less immobile pupil, while of the internal changes in the eye, the only one which need be mentioned here is the well-known glaucomatous cupping of the optic disc the weakest part of its walls and that which first or alone gives way and yields outwards before the pressure, from which ultimately results a true and incurable atrophy of the nervous filaments. The time varies immensely between the onset of the premonitory and, usually at first temporary symptoms, and the advent of visible organic change, but during this interval is *the* time to treat glaucoma, as it is *the* time to treat any other disease, and as no attack, however slight, leaves the eye as healthy as it found it, treatment can rarely be begun quite soon enough. It is luckily frequently the case that premonitory, slight and very temporary attacks warn the patient sometimes, at intervals, even for years, of his impending danger, and if he or his medical attendant is wise enough to take these seriously, the eye will have a prospect of continued utility, little impaired in comparison with what is likely to result from ill-advised procrastination. I need only refer to the remarkable results which have been achieved by operative measures for the cure of this disease—results which are limited only by the previous disorganization of the eye. But as regards treatment, my main desire is to throw out a warning against the indiscriminate use of mydriatics, especially atropine and cocaine, in affections of the eye. Glaucoma has often resulted from the use of belladonna, and is almost always accentuated by it; in most ocular diseases it is harmful, and the non-specialist would do well to cut this drug from his list in ophthalmic practice, except when convinced that he is dealing with an uncomplicated keratitis or an inflammation of the iris. On the other hand he has in myotics, and notably in eserine and pilocarpine, a fairly certain means of temporarily benefiting many cases of glaucoma, and so of saving valuable time till the most suitable method of treatment can be decided on. In connection with this subject, I can not perhaps do better in conclusion than say a word which may help to indicate how one can distinguish glaucoma from certain other affections *within* the eye with which it is sometimes confounded, as I have already referred to the frequency with which is mistaken for diseases without the eye.

The injection of the white of the eye in ordinary uncomplicated conjunctivitis is limited to the conjunctiva, and the engorged vessels move along with that membrane when it is rubbed over the sclerotic. The pupil also is unaffected.

The glaucomatous corneal haze might on a hurried examination be mistaken for superficial or interstitial keratitis, but the real corneal difference, the normal tension and condition of the pupil which is apt to be contracted in keratitis, should be sufficient guides.

In iritis there may be slight increase of tension, but the pupil is contracted, and the iris tissues are changed in color and in texture, while the anterior chamber retains its normal depth. Evidences of adhesion between the lens capsule and iris on dilation of the pupil will confirm the diagnosis. In one form of irido-cyclitis, with deep anterior chamber and deposit on the back of the cornea, there is fairly well marked increase of tension, but this is really a form of secondary glaucoma, requiring active treatment special to itself, into which I need not enter here.

Although in the olden days cataract was frequently confounded with glaucoma, there is no reason why it should be now that we know these to be essentially separate diseases. For the diagnosis of cataract one requires merely to observe the opacity in the lens, but it should be borne in mind that glaucoma may supervene in a cataractous eye; that primary cataract may happen to attack a glaucomatous eye, while secondary cataract is a very ordinary result of the long standing presence of glaucoma. It will be out of place and will take too long to discuss the difficulties attending the differential diagnosis between some chronic cases of glaucoma and optic atrophy, a subject attended by much difficulty, and concerning which there is great divergence of opinion. There is the less reason for such discussion in a paper which merely skims the surface of this subject, inasmuch as in these there is little likelihood that attention will be diverted from the eye, or the serious nature of the case lost sight of.

My object in this paper has been to present to you merely an outline of an important disease in such a form as to make it neither useless nor uninteresting, and my chief difficulty has been to avoid, on the one hand, leaving out important points, and on the other repeating what every one already knows. For mistakes in both directions I request your kind indulgence.

## Figures and Tables

**Figure f1:**